# Using ZIP Code and GIS Studies to Assess Disease Risk

**DOI:** 10.1289/ehp.10840

**Published:** 2008-01

**Authors:** Robert Golden, John D. Schell

**Affiliations:** ToxLogic, LLC, Potomac, Maryland, E-mail: rgolden124@aol.com; ENTRIX, Inc., Houston, Texas, E-mail: jschell@entrix.com

In several recent articles in *EHP*, investigators have attempted to link proximity to hazardous waste sites, as measured by geographic information systems (GIS) or ZIP codes, with increased incidences of various diseases. An implicit assumption in these studies is that proximity is a surrogate for exposure. However, numerous studies have demonstrated that the presence of chemicals in an individual’s environment does not necessarily translate into a dose. For example, Stehr-Green et al. (1988) found that

Serum PCB [polychlorinated biphenyl] levels in persons at highest risk of nonoccupationally related exposures … at 10 sites were within background ranges, even though environmental contamination levels as high as 2.5 parts per billion (ppb) in monitoring well water samples and 330,000 ppb in soil samples were measured.

In a recent study, Fitzgerald et al. (2007) investigated the impact of living near a known source of PCBs on the body burdens of local residents and concluded that

The results indicate no detectable differences in serum PCB levels according to proximity or wind direction relative to local point sources.

Consequently, it is erroneous to conclude that simply living in geographic proximity to a potential source is synonymous with an exposure, much less a dose, which is the critical determinant of risk.

The pitfalls of relying on spatial location as a surrogate for exposure and potential disease risk is illustrated by the study of Kouznetsova et al. (2007), which using ZIP codes reported an increased rate of hospitalization for diabetes and residential proximity to PCBs as a consequence of living near the Hudson River. The authors described this study as hypothesis generating, but nevertheless concluded that it provided “additional support for a relationship between exposure to environmental contaminants, especially POPs, and risk of diabetes.” We do not understand how a hypothesis-generating study (with no ability to account for a single known risk factor for diabetes) would offer support for an association between a chemical and a disease.

The hypothesis that PCB exposure might be etiologically involved in diabetes risk is not supported by the numerous mortality studies of PCB-exposed workers, none of which were mentioned by Kouznetsova et al. (2007). Table 1 summarizes diabetes mortality in all PCB-exposed occupational cohorts in which such data were reported. These data show no evidence that even prolonged occupational exposure to PCBs, with resulting accumulations approximately 100 times greater than background exposure, poses an increased risk of diabetes. It is reasonable to presume that if PCBs were etiologically implicated as a risk factor for diabetes, there would be increased mortality from diabetes in these cohorts. Because there is not, a more biologically plausible explanation for the findings of increased incidence of diabetes associated with environmental exposure to PCBs is that the accumulation and/or excretion of PCBs is a consequence of diabetes-related metabolic perturbations, not that diabetes is caused by PCBs.

## Figures and Tables

**Table 1:** Mortality from diabetes in PCB-exposed occupational cohorts.

Study	Mortality results for diabetes	Cohort size
Prince et al. 2006a	Total SMR = 0.88; 95% CI, 0.5–1.43 M SMR = 0.63; 95% CI, 0.17–1.61 F SMR = 1.02; 95% CI, 0.53–1.78	2,572 most heavily exposed M and F capacitor workers
Prince et al. 2006b	Total SMR = 0.89; 95% CI, 0.7–1.12 M SMR = 0.64; 95% CI, 0.40–0.98 F SMR = 1.06; 95% CI, 0.79–1.40	14,458 M and F capacitor workers
Ruder et al. 2006	Lowest tertile SMR = 0.42; 95% CI, 0.1–1.5	3,569 M and F capacitor workers
	Middle tertile SMR = 0.58; 95% CI, 0.1–1.7	
	Highest tertile SMR = 1.03; 95% CI, 0.3–2.4	
	Overall SMR = 0.67; 95% CI, 0.3–1.2	
Kimbrough et al. 2003	Hourly workers M SMR = 0.64; 95% CI, 0.29–1.21 F SMR = 1.19; 95% CI, 0.69–1.91	7,075 M and F capacitor workers
	Salaried workers M SMR = 0.79; 95% CI, 0.28–1.71 F SMR = 0	
Loomis et al. 1997	SMR = 0.56; 95% CI, 0.49–0.64	138,905 M electrical utility workers

Abbreviations: CI, confidence interval; F, female; M, male; SMR, standardized mortality ratio.
